# Evidence-based medicine self-assessment, knowledge, and integration into daily practice: a survey among Romanian physicians and comparison between trainees and specialists

**DOI:** 10.1186/s12909-020-1933-z

**Published:** 2020-01-16

**Authors:** Roxana-Denisa Capraş, Adriana Elena Bulboacă, Sorana D. Bolboacă

**Affiliations:** 1 Department of Medical Informatics and Biostatistics, Faculty of Medicine, “Iuliu Hațieganu” University of Medicine and Pharmacy, Cluj-Napoca, Romania; 2 Department of Anatomy and Embryology, Faculty of Medicine, “Iuliu Hațieganu” University of Medicine and Pharmacy, Cluj-Napoca, Romania; 3 Department of Pathophysiology, “Iuliu Hațieganu” University of Medicine and Pharmacy, Cluj-Napoca, Romania

**Keywords:** Evidence-based medicine (EBM), Survey, Mobile applications, Physician

## Abstract

**Background:**

A gap between the attitude towards evidence-based medicine (EBM), knowledge and awareness has been reported among physicians from different parts of the world. However, no investigation on Romanian physicians is available in the scientific literature. Our study aimed, firstly, to assess EBM awareness and the knowledge used by Romanian physicians, and, secondly, to compare resident trainees with specialists.

**Methods:**

Romanian trainee and specialist physicians were invited to participate in this cross-sectional study. The study tool was an online questionnaire designed to explore their awareness, knowledge, usefulness, the attitude in medical documentation, and the use of professional EBM resources. Data were collected by Google Form from January 1st to April 30th, 2017, respecting the responders’ anonymity. Two groups of physicians were investigated as trainees and specialists, respectively. Descriptive statistics (number, percentage, median and interquartile range) was used to describe the survey-related variables. Statistical significance on qualitative data was calculated with the Chi-square test, Fisher’s exact test, or the Z-test for proportions.

**Results:**

Two hundred and 50 physicians participated in this study (68% trainees vs. 32% specialists). In both groups, a significantly high percentage was represented by women as compared to men (trainees 72.4%, specialists 70%). The correct definition of EBM was identified by most respondents (75.6%). Affirmatively, both trainees and specialists always looked at levels of evidence when reading scientific literature, but a small percentage (6.5% trainees and 3% specialists) adequately identified the uppermost types of evidence in the hierarchy. Almost a quarter of the respondents shared the name of mobile EBM resources that they used to support the daily practice. Only six out of the 49 listed mobile resources met the EBM criteria.

**Conclusions:**

The participants proved to have limited knowledge of EBM and a positive attitude towards the concept. They made use of mobile medical resources without understanding which of these were evidence-based.

## Background

Evidence-based medicine (EBM) is the medical practice approach designed for optimizing decision-making by emphasizing the use of evidence supported by systematic and valid medical research. Evidence-based medicine starts from the premise that evidence of the highest quality, yielded from meta-analyses, systematic studies, and randomized clinical trials can offer accurate recommendations concerning individual medical decisions [1]. According to current definitions, EBM aims to render medical decisions more structured and objective, based on the clinical research results [2, 3]. This involves the use of guidelines developed based on results obtained from medical research, in the context of the clinical experience of the physician, and the patient’s desires and beliefs, in order to provide health care for individuals [4].

Aguirre-Raya et al. reported in 2016 the perception of EBM among medical students, interns, and specialists and reported a high global index of self-perception (75%), but this proved not to be supported by the global index on knowledge (19%) [5]. The gap between the attitude towards EBM and knowledge and awareness has been reported among physicians from different parts of the world (e.g., Saudi Arabia [6], United Arab Emirates [7], Ethiopia [8], Egypt [9], Japan [10], Belgium [11], Norway [12], France [13], or India [14]).

Medical applications that run on the smartphone could be an important source of information for EBM. Mobile medical applications emerged in 2008 and are available through dedicated stores (such as Apple Store, Google Play, Windows Phone Store, and BlackBerry App World) [15]. A study conducted in 2016 showed that just two out of 147 assessed EBM mobile applications running on Android, namely Medscape and DynaMed Plus, proved to be evidence-based [16].

Little is known about the clinical use of EBM in Romania. The few available articles are reviews [17, 18] or are limited to the evaluation of the knowledge gained after evidence-based training [19–21]. No articles were identified in the available scientific literature that presented the degree to which Romanian physicians managed to integrate evidence-based medicine into their daily practice.

Our study aimed to assess awareness, information and daily use of evidence-based medicine among Romanian physicians.

## Methods

### Study design

A cross-sectional and descriptive-analytical study was conducted from January 1st to April 1st, 2017.

### Participants

The participants in this study were physicians (medical trainees and specialists, regardless of their specialty) working in healthcare institutions in Romania. The graduates become trainees (or resident physicians) whenever they achieve at least 60% of the maximum score in the national examination (200 multiple choice questions answered within 4 hours). The residency period may vary from three to 7 years, depending on the specialty (e.g., 3 years for Family Medicine, 5 years for medical specialties, and up to 7 years for some surgical specialties such as Neurosurgery). A specialty exam ends the residency period and the trainees can opt not to do this exam and to start a new residency; these participants were considered in our study as trainees.

### Instrument

**Fig. 1:**
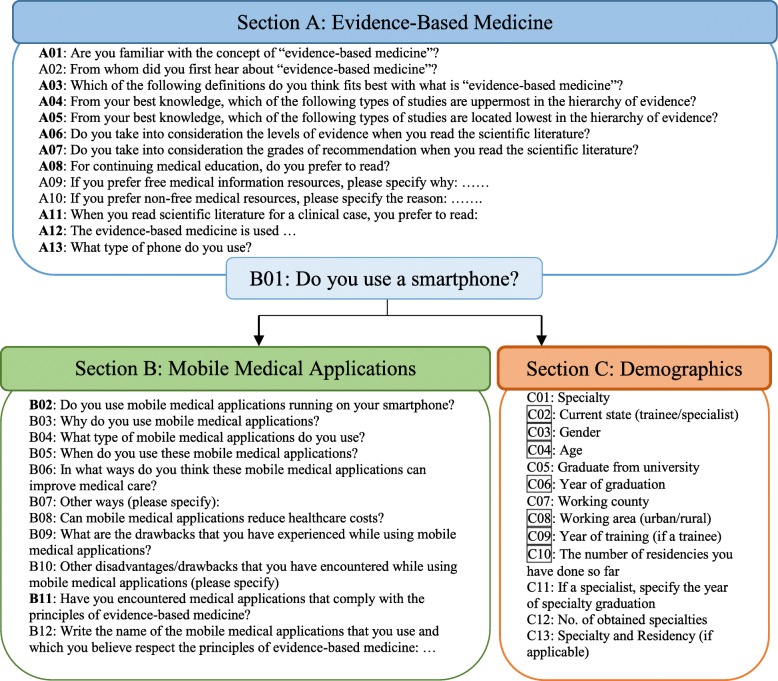
Content of the used questionnaire

The questionnaire consisted of three sections. Section A consisted of 13 questions referring to awareness, knowledge of evidence-based medicine, and knowledge of EBM resources. Section B consisted of 11 questions that referred to mobile medical applications (the use, advantages, disadvantages, identification of medical apps used that comply with EBM principles). Section C consited of questions referring to the socio-demographic characteristics of the respondents (Fig. 1).

Section B is available just for the users who choose “smartphone” as the answer in B01. Any other answer in B01 will lead the respondent to Section C. The answer of ten questions in Section A and two questions in Section B, along with the filling of Section C were required for valid submission for respondents who have an smartphone. The majority of the questions in the survey were closed, except for A09, A10, B07, B10, B12, C04 to C07, C10 to C13 that were open-questions (Fig. 1).

The classification systems for the quality assessment of the evidence formulated by the United States Preventive Services Task Force [22] were used in this questionnaire for the hierarchy of evidence and recommendation degrees.

The development of the survey and its validation were previously reported [23]. The validation of the questionnaire was carried out exclusively for Sections A and B. The items of the questionnaire, which were considered irrelevant, were not reported in this manuscript (Section C, namely C01, C05, C07, C11-C13, Fig. 1).

### Data collection

The online Romanian language version of the self-administered questionnaire, was promoted on professional groups on Facebook (see Additional file 1). All available Facebook groups of trainees and specialists as per January 1st, 2017 were selected for each specific specialty, including mixed groups that comprised physicians from various specialties in the country. A letter of intent, explaining the reason for joining the group and the purpose of the survey, was sent to each group administrator. Twenty-four professional groups consisting of about 75,117 members, with potential overlap (e.g., members who belonged to more groups) were used in order to ask the physicians to participate in this study. An invitation for participation was posted on each group, and a link to a Google Form was provided. At the beginning of the survey, the users were informed about the purpose of the study, the data collection procedure and anonymity (no personal data were collected to allow the identification of the respondent).

### Data analysis

A valid response was defined as the absence of an identical entry (identical answers to all questions in the survey) to avoid multiple participation.

The respondents were divided into two groups for analysis, namely: trainees and specialists. The respondents in training were considered trainees, irrespective of whether they were doing the first or second specialty.

Qualitative data were presented as numbers and percentages with associated 95% confidence intervals (provided in squared brackets along with the manuscript) [24] and were analyzed using the Chi-square test or Fisher’s exact test, as appropriate. Age was tested for normality with the Kolmogorov-Smirnov test, reported as a median and interquartile range, and compared with the Mann-Whitney test. Statistical significance was set at *p*-value < 0.05.

The mobile applications used and shared by the respondents were assessed to see whether they respected the evidence-based principles. Any apps that met the following three criteria were considered evidence-based medical applications: references supported the provided information, the degree of recommendation was specified, and the level of evidence was present.

## Results

### Characteristics of the participants

Data from 250 participants aged 24 to 63 were analyzed. The majority of respondents were, as expected, trainees. The majority of participants had only one specialty (211/250) and 14 participants were doing the second (13/250) and third specialty (1/250), respectively. The main characteristics of the respondents are presented in Table 1.

**Table 1:** Demographic characteristics and the specialty membership of the respondents

Category	Trainees (*n* = 170)	Specialists (*n* = 80)	*p*-value
Gender, no. (%)			0.70
Women	123 (72.4)	56 (70.0)	
Men	47 (27.6)	24 (30.0)	
Age, years median (Q1 to Q3) ^*^	27 (26 to 28)	40 (33 to 49)	< 0.0001
Work place, no. (%)			0.62
Urban	169 (99.4)	79 (98.8)	
Specialty, no. (%)			0.28
Medical ^a^	105 (61.8)	57 (71.3)	
Surgical ^b^	47 (27.65)	18 (22.5)	
Mixed specialties ^c^	18 (10.6)	5 (6.3)	

The values in the body of the table are represented by absolute frequency and percentage (the value in the round brackets) excepting the Age, for which the value of median and respectively first (Q1) and third (Q3) quartile is represented;

*p*-values are from Chi-Square test or Fisher’s exact test excepting *Mann-Whitney test.

^a^ Includes: Allergy and clinical immunology, Anaesthesia and intensive care, Infectious Diseases, Cardiology, Paediatric Cardiology, Dermato-venereology, Diabetes, nutrition and metabolic diseases, Endocrinology, Clinical Pharmacology, Gastroenterology, Paediatric Gastroenterology, Medical Genetics, Geriatrics and Gerontology, Haematology, Family medicine, Emergency medicine, Internal Medicine, Physical and Rehabilitation Medicine, Labour Medicine, Nephrology, Neonatology, Neurology, Medical Oncology, Paediatric Oncology and Haematology, Paediatric Pneumology, Paediatric Psychiatry, Psychiatry, Radiotherapy, Rheumatology, Pathological Anatomy, Epidemiology, Hygiene, Laboratory Medicine, Forensic Medicine, Nuclear Medicine, Radiology-Medical Imaging, Public Health and Management

^b^ Includes: General Surgery, Oral and Maxillofacial Surgery, Paediatric Surgery, Plastic Surgery, Thoracic Surgery, Vascular Surgery, Neurosurgery, Obstetrics and Gynaecology, Ophthalmology, Paediatric Orthopaedics, Orthopaedics and Traumatology, Otorhinolaryngology, Urology

^c^ Includes more than one medical, surgical or para-clinical specialty

The graduation year of the respondents varied from 1978 to 2016; most of the trainees graduated from the Faculty of Medicine 5 years prior to the year when the study was conducted (from 2012 to 2016; 77.6% [70.6 to 83.5]).

First year trainee physicians who participated in the study presented the highest percentage (35.8% [28.8 to 43.5]), while the smallest percentage was witnessed by trainees in the 5th (8 participants) or 6th (one participant) year of training.

### Evidence-based medicine assessment

The assessment of EBM self-evaluation and knowledge showed no differences between the respondents when the trainees were compared to the specialists (Table 2).

**Table 2:** Evidence-based medicine knowledge, awareness, and attitudes; numbers (percentage) reflect the results only for data from closed questions

Item	Trainees (*n* = 170)	Specialists (*n* = 80)	*p-*value
(A01) The concept of “evidence-based medicine”? ^a^			0.09
I have heard of it, but I do not know what it means …	17 (10.0)	2 (2.5)	
I know, I understand…, I am not using it…	27 (15.9)	10 (12.5)	
I know, I understand…, I am using it…	125 (73.5)	68 (85.0)	
I have never heard of it	1 (0.6)	0 (0.00)	
(A02) Source of information on EBM (multiple answers allowed)^b^			
Teachers	143 (84.1)	51 (63.8)	0.0004
Physicians	49 (28.8)	30 (37.5)	0.17
Colleagues	18 (10.6)	11 (13.8)	0.46
Nowhere	2 (1.2)	0 (0.00)	0.33
(A03) No. of correctly identified definitions ^c^	133 (78.2)	56 (70.0)	0.16
(A04) Uppermost in the hierarchy of evidence ^b^			
Systematic reviews and Meta-analyses	11 (6.5)	3 (3.8)	0.39
Systematic reviews	3 (1.8)	2 (2.5)	0.71
Meta-analyses	13 (7.6)	9 (11.3)	0.34
Randomized controlled trials (RCT)	12 (7.1)	9 (11.3)	0.27
Systematic reviews, Meta-analyses, RCT	19 (11.2)	7 (8.8)	0.05
(A05) Lowest in the hierarchy of evidence ^b^			
In vitro research	6 (3.5)	1 (1.3)	0.33
Animal study	1 (0.6)	1 (1.3)	0.57
Idea, editorial, or expert opinions	12 (7.1)	3 (3.8)	0.31
An animal research, in vitro research	5 (2.9)	1 (1.3)	0.44
Idea, editorial, review or expert opinions, animal research, in vitro research	10 (5.9)	0 (0.0)	0.03
(A06) Level of evidence considered when reading literature ^a^			0.21
Always	70 (41.2)	42 (52.5)	
Sometimes	72 (42.4)	31 (38.8)	
Never, but I know this classification	5 (2.9)	0 (0.0)	
I have ignored this classification	14 (8.2)	6 (7.5)	
I have never heard of it	9 (5.3)	1 (1.3)	
(A07) Strength of evidence ^a^			0.34
Yes, always	56 (32.9)	34 (42.5)	
Sometimes	77 (45.3)	36 (45.0)	
Never, but I know this classification	6 (3.5)	1 (1.3)	
I have ignored this classification	9 (5.3)	1 (1.3)	
I have never heard of it	22 (12.9)	8 (10.0)	
(A08) Continuing medical education: reading ^b^			
Online free medical articles	135 (79.4)	64 (80.0)	0.91
Online paid medical articles	58 (34.1)	38 (47.5)	0.04
Printed free medical articles	68 (40.0)	24 (30.0)	0.13
Printed paid medical articles	28 (16.5)	17 (21.3)	0.36
Medical articles available on apps	89 (52.4)	35 (43.8)	0.21
(A11) Reading scientific articles: preferences ^a^			0.28
Original articles	80 (47.1)	38 (47.5)	
Meta-analyses	49 (28.8)	18 (22.5)	
Systematic reviews	31 (18.2)	22 (27.5)	
I do not know the difference	5 (2.9)	0 (0.0)	
I do not read articles or journals; I prefer books	5 (2.9)	2 (2.5)	
(A12) The use of EBM ^b^			
Medical decisions	166 (97.6)	79 (98.8)	0.53
To know what is new in the medical field	104 (61.2)	34 (42.5)	0.01
To know the structure of a scientific paper	109 (64.1)	36 (45.0)	0.004
I have not yet understood the usefulness of EBM	3 (1.8)	0 (0.0)	0.23
I do not consider EMB in current practice	0 (0.38)	1 (1.3)	0.41

^a^ Fisher’s exact test; ^b^ Z-test for proportions; ^c^Chi-square test

The majority of respondents declared that they always looked at levels of evidence when reading scientific literature, but fewer than 6% adequately identified the uppermost types of evidence in the hierarchy (Table 2).

The financial aspect (39.2% [33.2 to 45.6]) and accessibility (easy and fast, 14.8% [10.8–19.59]) were the main reasons why the use of free sources of professional information was preferred. Sixty-eight of the respondents (27.2% [21.6–33.19]) pointed out that the accuracy of the information in paid scientific sources was higher as compared to free resources, but without providing any arguments. This result strictly reflects the perception of the respondents since the accuracy was not tested.

### Professional application usage on mobile phones

As expected, most of the respondents used a smartphone (96.8% [94.0 to 98.8]), with no difference between trainee and specialist respondents (*p*-value = 0.19). The characteristics of the medical apps used by the groups are summarized in Table 3. A statistically higher percentage of trainees (*p-*value = 0.001) used medical applications for disease aetiology (37/170), as compared to the specialists (4/80).

**Table 3:** Medical smartphone app usage as reflected by the closed questions

Item	Trainees (*n* = 170)	Specialists (*n* = 80)	*p*-value
(B02) The use of medical apps, yes ^a^	146 (85.88)	63 (78.75)	0.23
(B03) Reason for using … ^b^			
Curiosity	33 (18.24)	3 (3.75)	0.002
Allowing quick access to medical information	134 (78.82)	61 (76.25)	0.65
Most colleagues do it	4 (2.35)	0 (0.00)	0.17
To save time in identifying solutions to clinical problems	80 (47.06)	29 (36.25)	0.11
(B04) Top five type of medical applications ^b^			
Treatment	93 (54.7)	38 (47.5)	0.29
“Medical news”	79 (46.5)	30 (37.5)	0.18
Medical calculators	79 (46.5)	28 (35.0)	0.11
Diagnosis	61 (35.9)	21 (26.3)	0.13
“Scoring system”	55 (32.4)	22 (27.5)	0.43
(B05) When do you use the apps? ^b^			
for each patient, regardless of case	23 (13.5)	7 (8.8)	0.29
for atypical patients	64 (37.6)	31 (38.8)	0.86
to stay in touch with medical news	42 (24.7)	24 (30.0)	0.38
at home, for individual study	18 (10.6)	2 (2.5)	0.03
(B06) How can apps improve medical care? ^b^			
Fast search of scientific literature	135 (78.8)	56 (70.0)	0.13
Shortest time from consultation to diagnosis and treatment	61 (35.9)	19 (23.8)	0.06
Encouraging continuous medical documentation …	53 (31.2)	28 (35.0)	0.55
Availability to be used in any circumstance, even at the bedside …	33 (19.4)	13 (16.3)	0.56
(B08) Can mobile medical apps reduce healthcare costs? ^a^			
Yes, because they decrease unnecessary health service requests	50 (29.4)	23 (28.8)	0.92
Yes, because they avoid further expensive investigation	26 (15.3)	8 (10.0)	0.25
No	12 (7.1)	4 (5.0)	0.53
They could cut the future costs of healthcare …	113 (66.5)	51 (63.8)	0.68
(B09) Drawbacks in using medical apps ^b^			
incomplete definitions and treatment regimen	71 (41.8)	25 (31.3)	0.11
the absence of scientific references to support the evidence	87 (51.2)	26 (32.5)	0.01
an Internet connection is needed to access scientific articles	56 (32.9)	23 (28.8)	0.52
non-functional applications	47 (27.6)	12 (15.0)	0.03
they are limited to English speakers	26 (15.3)	9 (11.3)	0.40
the readability level is too low	34 (20.0)	9 (11.3)	0.09
(B11) EBM medical apps ^b^			
Explicit references, LE and DR	65 (38.2)	30 (37.5)	0.92
Only specified the LE	21 (12.4)	7 (8.8)	0.40
Only specified the DR	26 (15.3)	12 (15.0)	0.95
Only specified the references	21 (12.4)	10 (12.5)	0.98
I do not know mobile medical apps that implement EBM	19 (11.2)	3 (3.8)	0.06
I have not paid attention to this issue	8 (4.7)	0 (0.0)	0.05

The values in the body of the table are absolute frequencies and percentages corresponding to the sample size of each group; For this reason, the sum of percentages may exceed 100; *apps* Applications, *LE* Level of evidence, *DR* Degree of recommendation; ^a^ Chi-square test; ^b^ Z-test for proportions

**Fig. 2:**
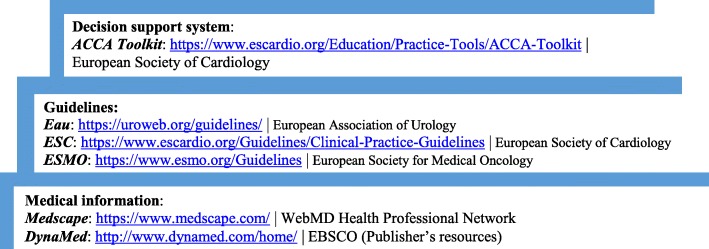
Mobile medical applications providing access to evidence-based medicine literature

A small number of respondents, 62 (24.8% [19.6 to 30.8]), chose to share the medical apps/online resources used in their daily practice or sources of lifelong professional education, resulting in a list of 49 distinct resources. The most frequently listed professional resource was Medscape (33 respondents, 53.22% [40.34 to 66.1]). Six (most of which are guidelines) out of the 49 listed resources provide access to evidence-based medical literature (Fig. 2). All six applications mentioned provide references, the level of evidence and degree of recommendation.

## Discussion

The results obtained in this study indicated a gap between the respondent’s EBM self-assessment perception and knowledge. This gap was identified in the youngest physicians (trainees) as well as in the specialists, without any differences between these two groups. However, the respondents have a positive attitude towards the EBM concept. The mobile medical resources are universally used by all respondents, but a small number of the listed mobile EBM resources is evidence-based.

### Knowledge, awareness, and attitudes

A significantly high percentage of women in the investigated sample reflects the gender distribution of graduates in Romania [25, 26]. A large percentage of the responders were trainees, which could be justified by the method of data collection, the trainees being more active on Facebook. Only 12% of all Romanian Facebook users were aged between 55 and 64 in 2017, while 22% of all Facebook users were aged 35 to 44 [27]. The interest of young physicians in evidence-based medical practice could also explain their participation in the study.

The investigated sample had a similar distribution of demographic characteristics of trainees and specialists regarding their workplace and specialty, with most of respondents having a medical specialty (61.8% of trainees and 71.3% of specialists). The predominance of medical specialties among respondents of EBM surveys was previously reported [12].

With few exceptions, trainees and specialists showed similar results regarding the EBM self-perception, knowledge, and attitudes towards evidence-based practice. In our sample, a significantly higher percentage of trainees received the information on EBM from their teachers. Similarly, a significantly higher percentage of trainees as compared to specialists were aware of the use of EBM as a source of knowledge and a way to know the structure of a scientific article.

Similar results were previously reported regarding the EBM self-assessment as well as the utility of evidence-based practice and the use of the EBM concept in the daily activity [5, 28]. Our study also reveals disagreement between how the respondents perceived their own EBM knowledge and their actual knowledge (Table 2, A01, A03, A04, A05). The knowledge had a direct impact on the decision-making process and could have a negative impact on clinical decisions. In our study, insufficient critical appraisal skills of a meta-analysis could explain the second place of meta-analysis in the list (Table 2, A04). Kitto et al. reported in 2007, in a small sample of surgeons, that personal experiences, one component of EBM, are seen as the best source of “evidence” [29].

It is widely recognized that accurate and valid scientific evidence is value-laden [30], but today’s evidence may turn out to be wrong tomorrow. The number of medical articles published in the scientific literature increases yearly and is also associated with the increasing number of retracted manuscripts [31] with misconduct as one of the chief causes of withdrawal, a severe issue to evidence-based practice [32]. Affirmatively most respondents consider the level (A06, Table 2) and strength of evidence (A06, Table 2) when they read scientific articles, but no reflection of knowledge related to the place of an article in the hierarchy of evidence (A04 and A05, Table 2) is observed. However, the classification of medical evidence strictly based on the type of the articles is superficial [33], and the training of physicians must be moved from the type of the manuscript to the quality of the article by assessing the applied methodology used to support the validity of the evidence. A meta-analysis classified as level A in the pyramid of evidence is useless if it proves to be of low or very low quality [34], and it is recommended not to be considered in medical decisions. The evidence-based practice training curriculum must be adapted to the changing nature of research evidence, to ensure a critical and deep understanding of the scientific literature but not based on a hierarchy according to the type of article.

### Medical applications and professional resources

Similarities between trainees and specialists were observed regarding the type of medical apps, regarding the situations when these tools were used, and the perception vis-à-vis to their usefulness in healthcare. There were also differences, though. Compared to specialists, trainees had a significantly more frequent use of medical apps out of curiosity and for individual professional study at home. A more frequent use of the disease aetiology apps by trainees, as compared to specialists, which was identified in our study, could be explained by the trainees’ lack of professional experience. Furthermore, a higher percentage of trainees pointed out the absence of scientific references to sustain the evidence and the non-functional application as the main drawbacks of medical apps. Some of the differences may be related to the specialists drawing on higher clinical expertise and clinical pattern recognition, both of which being parts of an evidence-based practice approach. The absence of scientific references and use of non-functional apps by specialists could be explained by the education received by trainees as well as by their eagerness of trying more applications. A minimal number of the medical apps listed by the respondents met the EBM criteria (6/49).

Medical apps have brought many benefits to the medical field, namely: chiefly faster decisions, with lower error rates, higher quality medical information, accessibility to it [35], and support in clinical decision-making at the place of treatment [36]. Medical apps have automatic updates as a main feature and allow fast access to up-to-date scientific literature [37, 38]. Medical apps could also employ standard formulas to make calculations and determine risks, such as body mass index (BMI), body surface area (BSA), or adequate drug dosage [35]. More complex mobile apps serve as a diagnostic tool in migraines by identifying the triggering factors; they also facilitate the patient-doctor contact during migraine attacks, and assist the management of treatment strategies [39]. Medical apps can help doctors to offer personalized initial antidepressant drugs according to the patients’ symptoms, conditions and other medications [40]. The use of medical apps has also been increased in specialities where clinical observation is a vital diagnostic tool such as Dermatology, Ophthalmology or Radiology [41–43]. Furthermore, the usefulness of smartphone apps in telecare was also reported, especially in the long-term management of diseases, with increase in medication adherence and easy patient-doctor communication, especially in the case of initiation of new treatments [44]. Medical apps started to be an important part of clinical medicine, helpful for patients and doctors alike.

### Limitations, implications, and perspectives

The main limitation of our study is related to its design. Several issues could be listed, related to the sampling method, data collection, and the content of the survey. Firstly, a non-probabilistic method was used in our study to identify the sample, so the results obtained need to be carefully interpreted. According to the physician medical code, a probabilistic sampling method, such as the simple or stratified by age random method, could be a better solution in order to identify the respondents. Hence, this will closely reflect the population of Romanian physicians, allowing the generalizability of the results. Secondly, the use of Facebook groups as a method to invite the target population to participate in the survey could induce a selection bias, and thus, the investigated sample is not necessarily representative for the Romanian physicians. The largest demographic groups of Facebook users worldwide are represented by 18–27 - year - old women, and 25–34 - year - old men, respectively [45]. In this context, the method used in our study for inviting the participants limits the possibility to reach the senior physicians.

Consequently, the reported results have a limited impact on the population of Romanian physicians, thus the behaviour of young physicians outweighing the actions of senior physicians. The use of the random sampling method by considering all Romanian physicians and sending a printed survey by post or an individual e-mail invitation with recall could engender a more realistic picture of the use of EBM in their daily practice by Romanian physicians. Thirdly, the survey that was used evaluated the respondents’ EBM knowledge and the self-perception behaviour in connection with the use of medical evidence, either printed or online. A more in-depth assessment based on problem-based scenarios would allow a reli able assessment of the EBM knowledge and their application in clinical practice. Furthermore, since the “survey approach is a research strategy, not a research method”, it was intended to evaluate “how things are at a specific time” [46], and thus had its limitations, such as the lack of details and in-deep analysis, the fact that the respondent could provide a predictable answer, the lack of control over the person who actually accesses the survey link, and the possibility of arbitrary answers [47]. A further limitation could be grouping, definition and subsequent analysis of trainees, who were defined as those currently in training at the time of the questionnaire completion, irrespective of whether previously being trainees or having completed one or more specialties. However, since just only 14 participants were in this situation, this was not expected to significantly influence the results.

### Strengths

Despite its limitations, our study has had several characteristics of value. Firstly, our results provided a snapshot regarding EBM self-assessment and knowledge in the group of respondents. Our result supports the need for evidence-based practice training at the level of undergraduate students, trainees as well as specialists. The onsite or online courses, even during scientific meetings and conferences, with the translation of EBM information/knowledge into problem-based learning, could ensure a better understanding of the concepts. The shift from the use of evidence based on the type of the article to the use of medical evidence based on their quality is a must and could be done through more thorough EBM training. Integrating EBM in the daily health care practice could serve as a supplementary resource for knowledge, in addition to guidelines, which are very helpful for clinical decision-making whenever a specific case not covered by the clinical guidelines needs to be managed.

## Conclusions

Most of the participants in our study were able to identify the correct definition of evidence-based medicine but failed to recognize the levels of evidence correctly. The participants’ attitude towards EBM was positive. Mobile medical apps were used in the daily clinical activity, but the respondents could not differentiate between the apps, which comply and those which do not comply with EBM principles. The changing nature of research evidence supports the move beyond the EBM training to quality assessment regardless of level, grade or evidence.

## Supplementary information

**Additional file 1.** List of Facebook groups invited to participate in the study.

## Data Availability

The raw data supporting the conclusions of this article and the translated questionnaire are available on request, from the corresponding author.

## Abbreviations

App(s): Application(s)
BMI: Body mass index
BSA: Body surface area
DR: Degree of recommendation
EBM: Evidence-based medicine
LE: Level of evidence
RCT: Randomized controlled trials
