# Supplementary material for: Evidence-based medicine self-assessment, knowledge, and integration into daily practice: a survey among Romanian physicians and comparison between trainees and specialists
Source: BMC Med Educ. 2020 Jan 16;20:19. doi: 10.1186/s12909-020-1933-z (PMC6966825; doi:10.1186/s12909-020-1933-z)
Supplement: Supplementary file 1 — Additional file 1. List of Facebook groups invited to participate in the study. [file 12909_2020_1933_MOESM1_ESM.docx]

Facebook groups invited to participate at the study. The members of members as per January 1^st^, 2017.

| **Facebook group name** | **https://www.facebook.com/groups/** | **Members** |
| --- | --- | --- |
| [Doctors ready for anything! ;-) Be smart, be open, be a doctor, be you!]  Medici gata de orice! ;-) Be smart, be open, be a doctor, be you! | 1777799889131284/ | 2,852 |
| [Physicians ready for general strike. We request decent salaries!]  Medici gata de greva generală. Solicitam salarii decente! | 1667018620201805/?ref=br_rs | 34,160 |
| [General Surgery Trainees Bucharest 2015]  Rezidenti Chirurgie Generala Bucuresti 2015 | 1619783204953159/ | 120 |
| [Doctorate/PhD UMF "Iuliu Hatieganu" Cluj]  Doctorat/PhD UMF "Iuliu Hatieganu" Cluj | 1057812207571859/ | 191 |
| [Medical Oncology Trainees (2015-2020)]  Medici rezidenti de oncologie medicala (2015-2020) | 904946579530028/ | 81 |
| [Trainees of Internal Medicine Sibiu]  Medici rezidenti de Medicina Interna Sibiu | 771632732860732/ | 170 |
| [Vascular Surgery Cluj]  Chirurgie Vasculara Cluj | 897000183722645/ | 30 |
| [Târgu-Mureș Trainees]  Rezidenti Targu-Mures/Marosvasarhelyi Rezidensek | 749087828517657/ | 952 |
| [Doctors and Pharmacists in Romania]  Medici si farmacisti din Romania | medfarmro/ | 13,959 |
| [Obstetrics and Gynecology Cluj-Napoca]  Obstetrica- Ginecologie Cluj- Napoca | 576631005771638/ | 18 |
| [Psychiatry Trainees Cluj-Napoca]  Medici Rezidenti Psihiatrie Cluj-Napoca | 545223035585118/ | 158 |
| [Specialist Physicians]  Medici specialisti | 480238968804388/ | 182 |
| [Physicians and Trainees]  Doctori si Rezidenti | 697912936912209/ | 8,235 |
| [Pediatric Trainees Cluj]  Rezidenti Pediatrie Cluj | 347200355459397/ | 36 |
| [Residency 2014]  Rezidentiat 2014 | 440525646053343/ | 6,491 |
| [Galați Trainees]  Rezidenti Galați | rezidenti.galati/ | 125 |
| [Cardiology Trainees]  Rezidenti cardiologie | 400144803393053/ | 137 |
| [Family Physicians from Romania on Facebook]  Medici de familie din Romania pe Facebook | medicidefamilieromani/ | 4,554 |
| [Obstetrics and Gynecology Trainees]  Rezidenti obstetrica-ginecologie | 295199253847290/ | 527 |
| [Trainees Physicians Timișoara]  Medici rezidenti Timișoara | 292872917588093/ | 756 |
| [Constanța Trainees]  Rezidenți Constanța | 244035462327198/ | 450 |
| [Trainees Ophthalmologist]  Medic Rezident Oftalmologie | 387759984646428/ | 498 |
| [Trainees physicians - Oradea]  Medici rezidenți - Oradea | 250085115172596/ | 135 |
| [Pneumology Trainees Group]  Grupul Medicilor Rezidenti Pneumologi | PneumoGroup/ | 300 |
